# Contribution of preclinical MRI to responsible animal research: living up to the 3R principle

**DOI:** 10.1007/s10334-021-00929-w

**Published:** 2021-05-19

**Authors:** Lydia Wachsmuth, Armand Mensen, Cristina Barca, Marlene Wiart, Catarina Tristão-Pereira, Alice Busato, Sonia Waiczies, Uwe Himmelreich, Jason M. Millward, Henning M. Reimann, Ileana Jelescu, Pasquina Marzola, Bruno Pradier, Angèle Viola, Cornelius Faber

**Affiliations:** 1 Translational Research Imaging Center, Clinic for Radiology, University Hospital Münster, Albert-Schweitzer Campus 1, 48163 Muenster, Germany; 2 Swiss 3R Competence Centre, University of Bern, Hochschulstrasse 6, 3012 Bern, Switzerland; 3 European Institute for Molecular Imaging (EIMI), University of Münster, Waldeyerstraße 15, 48149 Munster, Germany; 4 Univ-Lyon, CarMeN laboratory, Inserm U1060, INRA U1397, Université Claude Bernard Lyon 1, INSA Lyon, Charles Mérieux Medical School, 69600 Oullins, France; 5 Animal Imaging and Technology, EPFL, Station 6, 1015 Lausanne, Switzerland; 6 CIBM Center for Biomedical Imaging, EPFL, Station 6, 1015 Lausanne, Switzerland; 7 Centro Nacional de Investigaciones Cardiovasculares, Calle de Melchor Fernández Almagro, 3, 280291 Madrid, Spain; 8 Department of Computer Science, Strada Le Grazie 15, 37134 Verona, Italy; 9 Berlin Ultrahigh Field Facility (B.U.F.F.), Max Delbrück Center for Molecular Medicine in the Helmholtz Association, Robert-Roessle-Straße 10, 13125 Berlin, Germany; 10 Biomedical MRI, Department Imaging and Pathology, KU Leuven, Herestraat 49, bus 505, 3000 Leuven, Belgium; 11 Aix-Marseille Univ, CNRS, CRMBM UMR 7339, Faculté des Sciences Médicales et Paramédicales la Timone, 13005 Marseille, France

The contribution of preclinical MRI in advancing 3R efforts in the life sciences is undoubtedly a hot area of discussion. For this reason, the 2nd ESMRMB preclinical MRI day “Frontiers in Preclinical MRI” on September 30, 2020 was included in the program of the online Annual Meeting of the ESMRMB and was focused on animal welfare. A dedicated two-hour session: “*Living up to the 3R principle—Contribution of preclinical MRI*” provided the forum for discussion about contributions of preclinical MRI to the 3R efforts in the life sciences. After setting the stage with a 30-min introductory lecture about the 3R principle by Armand Mensen from the Swiss 3R Competence Centre, nine selected contributions that were received in response to a Europe-wide call on the topic, provided insight into capabilities of MRI, its current use and shortcomings that must be addressed in the future.

## General considerations on ethics in experimental animal research

In 1959, William Russel and Rex Burch [1] proposed that in the interest of laboratory animal welfare, scientists should adhere to the recommendations of the 3Rs: *replacement, reduction, refinement*. This topic was elaborated upon during the introductory lecture. Since Russell and Burch, 3R recommendations have evolved into requirements and animal welfare legislation that are imposed upon the researcher. Accordingly, the most referenced EU Directive (Directive 2010/63/EU on the protection of animals used for scientific purposes) states that animals have intrinsic value that needs to be respected. Animal welfare considerations should be given the highest priority as per the directive, and each use needs to be carefully evaluated. All in all, the 3Rs should be considered systematically when using animals in biomedical research.

*Replacement* stands for making sure that research questions cannot be answered by alternative approaches using material from non-living animals, such as cell culture, 3D organoids, or multiple organ systems; or that animal experiments cannot be replaced by developing computational models. Furthermore, successful replacement can also be accomplished when using creatures considered to be less prone to the consequences of suffering (based on the phylogenetic hierarchy). Such creatures should be selected for experiments (e.g. invertebrates instead of vertebrates) whenever appropriate to the experimental goals. *Reduction* is the R where non-invasive imaging excels best due to the stark reduction in required animal numbers. With longitudinal monitoring, comparable levels of information can be retrieved from fewer animals. Progression of disease or treatment response can be tracked, statistical power is increased, and individual trait differences (for example early versus late responders) can be identified. Non-invasive imaging also allows the retrieval of *more* information from the same number of animals. Preclinical MRI offers multiple techniques to assess numerous outcome measures within one MRI session. Additionally, MRI may be complemented by further non-invasive imaging techniques (e.g. PET or optical imaging) and by behavioral and clinical examinations. Armand Mensen extended the general narrow focus of the reduction principle introducing concepts which improve experimental design by considering for example sex bias and standardization fallacy [2, 3]. *Refinement* of experimental techniques to minimize or alleviate pain, suffering and distress not only serves animal welfare but also improves science considerably. Environmental enrichment has a positive impact on animal stress, anxiety and behavior as well as scientific results. Reducing unnecessary stressors can lead to more reliable and robust results [4]. Impressive in this regard is research on the impact of non-aversive handling [5, 6].

**Fig. 1:**
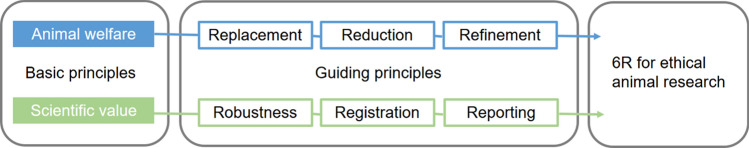
Two basic principles for experimental animal research translate into six practice-guiding principles (6R). [Modified from [7]]

The 3R principle now refers to far more than each of the Rs mere classical definition. In addition to ethical aspects, the scientific value of research with animal welfare measures is of utmost importance. In a recent publication, Strech and Dirnagl [7] proposed to complement the ethical 3Rs with a set of measures assuring scientific quality of animal experiments, namely *robustness, registration and reporting* (Fig. 1). These additional principles demand sample size calculations and blinding to enhance the validity and reliability of results. Pre-registration of working hypotheses and experimental designs in public registries [8] together with extensive reporting (for example according to the Animal Research: Reporting of In Vivo Experiments (ARRIVE) guidelines [9]) are suggested to increase transparency and reproducibility and to justify the relevance of the proposed experiments.

## Contributions of preclinical MRI to the 3Rs

Following the introductory lecture, nine presentations illustrated exemplary applications of MRI in preclinical studies in the context of the 3R principle. Given the non-invasiveness of MRI and the option to perform longitudinal studies, several examples highlighted the contribution of preclinical MRI to reduce animal numbers.

Cristina Barca et al. presented a longitudinal, multimodal imaging study in a mouse stroke model [10]. T2-weighted anatomical MRI was combined with diffusion-weighted MRI and arterial spin labeling MRI to assess brain perfusion. At alternating time points, MRI was complemented by DPA PET to measure inflammation. By repeated examinations in individual mice, animal numbers could be reduced from 96 to 16 in this study.

Marlene Wiart et al. shared data from another preclinical stroke trial in rats that evaluated the neuroprotective effects of remote ischemic conditioning [11]. The primary endpoint was edema-corrected lesion size measured by T2-weighted MRI at 24 h of reperfusion normalized by the area-at-risk of infarction determined from per-occlusion MRI for each individual. Of note, with T2-weighted anatomical MRI lesion size was more sensitively detected compared to TTC (2,3,5-triphenyltetrazolium chloride) staining of fresh brain tissues, the gold standard of reference for evaluation of infarct size in conventional experimental animal studies. Sample size calculations based on the effect sizes in this study revealed 19 instead of 55 animals per group when planning a longitudinal MRI study instead of using TTC staining of fresh brain tissues, the gold standard for evaluation of infarct size in conventional experimental rodent studies. Including the 40% exclusion rate (due to mortality but also to the strict inclusion of animals that have effectively experienced brain ischemia as documented by per-occlusion MRI) this resulted in 64 versus 184 surgeries, or 120 spared animals. The fact that in the clinical setting lesion size is usually determined by MRI highlights the outstanding translational value of MRI-based diagnostic endpoints in preclinical stroke studies.

Catarina Tristão-Pereira et al. presented a longitudinal multimodal imaging study combining resting-state fMRI, diffusion-weighted MRI and FDG-PET in a rat model of Alzheimer’s disease. The pathology of progressive neurodegeneration induced by brain glucose metabolism disruption in this animal model (effectively “type 3 diabetes”) had previously only been characterized at different timepoints using histology [12], thus recruiting a larger number of animals while not benefiting from a truly longitudinal—but rather cross-sectional—analysis. Here, animal numbers were reduced by a factor of three and the study reported a non-monotonic trend in white matter degeneration and changes in resting-state functional connectivity characterized by an early acute decline, temporary recovery and finally chronic degeneration [13] which preceded a similar trend at the behavioral level [12]. Combination of several complementary imaging methodologies at the same time increased the scientific value of the study. Furthermore, the resting-state fMRI and anatomical MRI data are publicly available for reuse: https://openneuro.org/datasets/ds003520. The use of medetomidine over alpha-chloralose for the fMRI studies also decreased animal count.

Alice Busato et al. presented a MRI investigation of their transgenic mouse model of pancreatic cancer (KPC). High-resolution T2-weighted images were acquired longitudinally, starting from 3-month-old mice, every 10 days, until the humane endpoint. MRI was able to follow the evolution of the four-class imaging-based tumor staging, detecting the first stage of the tumor as multiple liquid cysts inside pancreas (hyper-intense alterations), and its evolution toward a solid mass in a single subject. Due to the longitudinal study design, animal numbers were substantially reduced. Moreover, at the last stage of the pathology, MRI allowed detection of metastasis in the abdominal region.

All 3R principles, replacement, reduction and refinement, were met in a study by Liesbeth Vanherp et al. in a murine model of cerebral *Cryptococcus neoformans* infections. Typically, virulence is studied by infecting large groups of animals and recording survival or analyzing fungal burden of the infected organs ex vivo, which does not provide insights in how disease establishes and progresses. With MRI, already disease onset was indicated by enlargement of ventricles, edema, hydrocephalus, presence of parenchymal lesions, and fluid accumulation around major vasculature and the meninges. Even when survival rates were similar, the relative contributions of these different parameters were highly variable between strains, but not between animals infected with the same strain. By assessing animals repeatedly (up to seven times) over a period of up to 39 days after infection, this MRI-based in vivo virulence study reduced animal numbers by at least a factor of six when compared to an exclusively histology-based approach. By providing early virulence markers, preclinical MRI could replace pure survival rate studies. Refinement could also be addressed, because burden of the animals would substantially be reduced by sacrificing them before severe symptoms cause their death.

Pathological observations in animal models of disease are of little consequence if they do not reflect the human counterpart. Millward et al. observed dynamic expansions and contractions of ventricle volumes in the mouse model of multiple sclerosis (experimental autoimmune encephalomyelitis) by repeated MR measurements over a 2-month period [14]. In Multiple Sclerosis (MS) patients, short-term fluctuations in ventricle volumes were less striking than those in the animal model but they were clearly present. Interestingly, MS patients showing expansions and contractions beyond the normal range of variation were at an earlier disease stage. This suggests that fluctuations are indicative of inflammatory activity. The preclinical study facilitated a significant reduction in animal numbers. Pathological observations were relevant to MS patients and could be of future benefit in the diagnosis and monitoring of MS patients.

In summary, these examples showed that considerable reductions in animal numbers were accomplished by repeatedly imaging animals during the course of the experiment, instead of running *verum* and control groups for each time point. This allowed for investigating dynamic processes, like disease progression or treatment response. Each animal served as its own control/baseline and trait differences could be tracked individually. Statistical power was higher compared to inter-individual comparisons. Integrating multiple imaging modalities (for example different MRI methods and/or PET) and complementing study design by behavioral measures, clinical examinations and histological validation increased the scientific value of these studies. Translational value in particular of MRI endpoints was high.

Two further contributions showed examples where MRI contributed to the 3R principle of replacement.

Gordon Winter et al. introduced the use of hen’s egg test chorioallantoic membrane model (HET-CAM) in MRI for in vivo testing of novel target-specific radioligands [15]. Multi-modal PET and MR imaging of hen’s eggs before embryo development day 20 represent an alternative to established small animal models for biodistribution assessment. Only successful candidates then proceed to further testing for example in the severe combined immuno-deficient (SCID) mouse model.

Millward et al. shared their method for preparing fixed rodent phantoms for ex vivo MR imaging [16]. Such phantoms preserve the internal anatomy in situ. They are useful for introducing trainees into pre-clinical MRI of various target organs, without the need for living animals. The phantoms can be conveniently stored and re-used, reducing the total number of animals needed. The phantoms are particularly useful for developing and refining new MRI methods, thus improving the reliability and reproducibility of subsequent imaging experiments with living animals.

Finally, with “OpenMind”—The Open Database of Anesthetic Effects and Protocols for Preclinical Research Henning M. Reimann et al. presented an example on how MRI complies with the extended 3R principles. Rodent functional magnetic resonance imaging (fMRI) can replace more invasive readouts for assessing brain activity. However, the necessity to anesthetize animals during examination has a prominent impact on study outcome and comparability with human studies. A recent publication by the group [17] outlined that anesthesia affects overall physiology, including brain states, functional networks, information transfer, sensory processing and hemodynamic integrity—i.e., the physiological translation of neuronal activity into hemodynamic responses captured by fMRI. Thereby, different anesthetics affect the above measures in distinct ways, which pose a further challenge for data interpretation. An open encyclopedia for anesthetic effects is proposed as platform to report and share findings and data with the community. The goal is to provide a powerful web-based infrastructure for fast and easy dissemination of findings, data, and protocols to gain a better understanding of anesthetic effects. This will help to establish optimized anesthetic protocols and define robust experimental conditions for functional neuroimaging studies, and in turn foster scientific quality and reduce animal numbers.

While there is always more to be done, the interactive panel and attendees agreed that preclinical MRI indeed is living up to the 3R principle. Foremost, the aspect of reduction is addressed by the possibility to perform longitudinal studies allowing precise monitoring of disease progression and response to therapy in models of different diseases. Biological variability can be alleviated, as each animal serves as its own control. Added scientific value is provided by simultaneously acquiring molecular, functional, metabolic and anatomical information and by the combination of complementary non-invasive imaging modalities. Such combinations are feasible, since the Directive 2010/63/EU states only minor burden experienced by the animals, mostly due to the impact of anesthesia (length of examination times and number of repeated anesthetic interventions). However, multiple longitudinal measures of an animal can act as an additional stressor in and of itself as it involves an increase in experimenter handling. However, these can be mitigated using appropriate refinement techniques, such as tunnel handling and environmental enrichment approaches. Its non-invasiveness makes preclinical MRI a possible alternative to replace conventional, invasive experimental techniques. The fact that MRI is the established diagnostic method in the clinical setting for many diseases adds high translational value. However, MRI needs to mature to a “gold standard” for outcome parameters.

## Steps to be taken to exploit the full potential of MRI with regards to the 3R principle

**Fig. 2:**
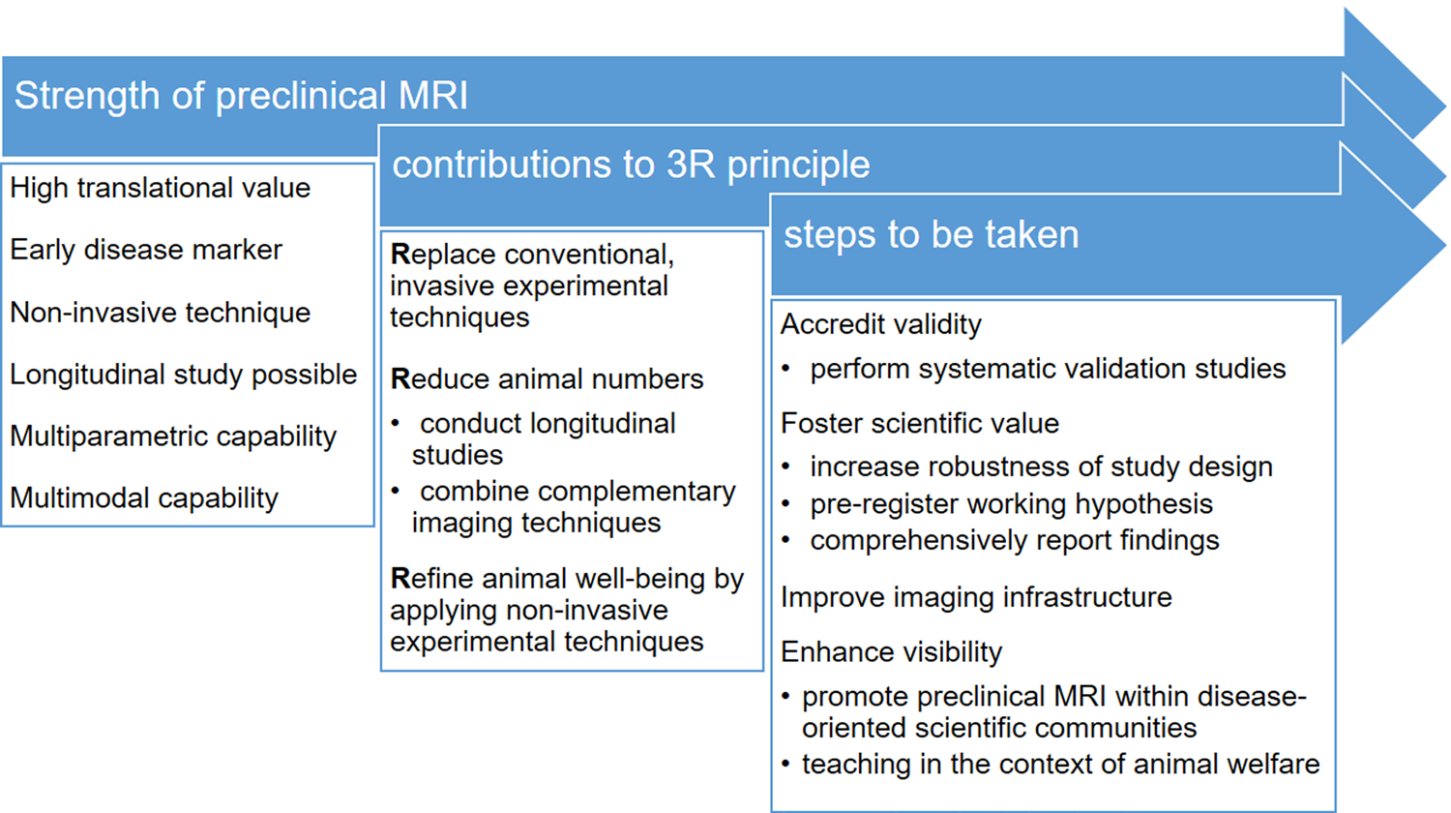
Preclinical MRI within the framework of the 3R principle

The general appraisal of the attendees of this session was that within the imaging community there is little doubt about the merit of MRI in experimental preclinical research, but its potential is not yet fully tapped. Within the framework of the 3Rs (Fig. 2), preclinical MRI contributes to Reduction of animal numbers and allows for Refinement or even Replacement of more invasive experimental techniques. However, MRI metrics alone are often not considered sufficient by reviewers and funding agencies, and more invasive experiments are frequently requested.

What can be done to raise the awareness of the value of MRI and to establish it as reference standard? Obviously, systematic validation studies are needed to accredit validity and to convince the scientific community that MRI-based endpoints often outperform conventional, invasive outcome measures, as shown by examples of determining lesion size in stroke models or tumor volume in solid cancer imaging. In particular, MRI measures win over as early disease marker for many applications. Adhering to the extended 3R guidelines, Robustness, Registration and Reporting will further strengthen the scientific value and enhance acceptance of preclinical MRI. Other important initiatives in this regard are efforts to implement standardized animal imaging protocols, for example for whole-brain fMRI [18]. However, even if the added value of preclinical MRI has been widely accepted, it is often not employed as tool to provide valid data, but only represents the cherry on the methodological cake. One reason why non-invasive imaging still has not yet become a routine application may be the location and infrastructure of most imaging facilities (availability of the MRI systems, cost of MR sessions). Substantial regulatory work is often required before animals can be transferred to an imaging facility. Hygiene requirements may prohibit that animals leave the imaging facility once they have entered it, and additional experimental options, such as behavioral testing or surgical intervention, may not be available in the imaging facilities. To exploit the potential of performing longitudinal studies, sufficient animal housing options and room for performing complementary experimental procedures should be integrated within imaging facilities whenever possible.

Efforts by the whole preclinical imaging community are required to promote MRI and other non-invasive imaging methods. Leaving the comfort zone of discussing preclinical MRI studies within the imaging community, and advocating imaging studies more proactively in front of clinicians and disease-oriented biomedical research communities will help to promote preclinical MRI.

Further measures include engaging in work with the animal welfare authorities, especially in local commissions where individual experiment licenses are discussed. Also getting involved in animal welfare training within the scope of Federation of European Laboratory Animal Science Associations (FELASA) may raise the community’s recognition of the value of non-invasive preclinical MRI and promote its routine application in experimental animal research in line with the 3R principle.
